# KMT2A histone methyltransferase contributes to colorectal cancer development by promoting cathepsin Z transcriptional activation

**DOI:** 10.1002/cam4.2226

**Published:** 2019-05-15

**Authors:** Yang Fang, Dan Zhang, Tingting Hu, Hongyan Zhao, Xuan Zhao, Zhefeng Lou, Yongshan He, Wenzheng Qin, Jianfu Xia, Xiaohua Zhang, Le‐chi Ye

**Affiliations:** ^1^ Zhejiang Provincial Key Laboratory for Technology and Application of Model Organisms, Key Laboratory of Laboratory Medicine Ministry of Education Wenzhou China; ^2^ School of Laboratory Medicine and Life Sciences Wenzhou Medical University Wenzhou China; ^3^ Department of Respiratory Medicine The First Affiliated Hospital of Wenzhou Medical University Wenzhou China; ^4^ Department of Gastroenterology and Hepatology The First Affiliated Hospital of Wenzhou Medical University Wenzhou China; ^5^ Department of Colorectal and Anal Surgery Xinhua Hospital, Shanghai Jiao Tong University School of Medicine Shanghai China; ^6^ Endoscopy Center Zhongshan Hospital, Fudan University Shanghai China; ^7^ Department of General Surgery Wenzhou Central Hospital Wenzhou China; ^8^ Department of Colorectal and Anal Surgery The First Affiliated Hospital of Wenzhou Medical University Wenzhou China

**Keywords:** cancer development, CTSZ, epigenetic modifier, KMT2A

## Abstract

Accumulating evidence supports the notion that epigenetic modifiers are abnormal in carcinogenesis and have a fundamental role in cancer progression. Among these aberrant epigenetic modifiers, the function of histone methyltransferase KMT2A in somatic tumors is not well known. By analyzing KMT2A expression in patient tissues, we demonstrated that KMT2A was overexpressed in colorectal cancer tissues in comparison with adjacent normal tissues and its expression was positively correlated with cancer stages. In KMT2A‐knockdown HCT116 and DLD1 cells, cell invasion and migration were consequently suppressed. In addition, KMT2A depletion effectively suppressed cancer metastasis in vivo. Mechanistically, cathepsin Z (CTSZ) was demonstrated to be an important downstream gene of KMT2A. Further studies showed that p65 could recruit KMT2A on the promoter region of the downstream gene CTSZ and knockdown of p65 could reduce the KMT2A on the promoter of CTSZ. Finally, our present study revealed that KMT2A epigenetically promotes cancer progression by targeting CTSZ, which has specific functions in cancer invasion and metastasis.

## 1 INTRODUCTION

Accumulating evidence indicates that many histone modification enzymes play a critical role in cancer development and might be important drug targets. Histone H3 lysine 4 (H3K4) methylation has several distinct functions in gene expression regulation,^1^ and is modified by specific methyltransferases and demethylases.^2^ In detail, H3K4 methylation can be mono‐, di‐, or tri‐methylation (H3K4me1/2/3). For example, H3K4me3 is highly enriched at gene transcription start sites and actives gene transcription. Therefore, many cancer‐related genes regulate transcription initiation by adding or removing covalent modifications on histones. The KMTs family contains the SET domain that methylates H3K4 and can promote genome accessibility and transcription.^3^ There are three pairs of KMTs members: KMT2A/KMT2D, KMT2C/KMT2B, SETd1A/SETd1B, which all play important roles in cacinogenesis, cancer progression and prognosis. KMT2A is the crucial member of the KMTs family and can trimethylate H3K4.^4^ Although misregulation of KMTs family proteins has been associated with aggressive lymphoid and myeloid leukemias, the putative biological function of KMT2A in the colorectal cancer (CRC) is not well defined.

Cathepsin Z (CTSZ), is a cathepsin family member and located in a frequently amplified region at 20q13.2.^5^ It contains an Arg‐Gly‐Asp (RGD) motif, which is cleaved and removed following its activation.^6^ RGD is a sequence that is important for the protein to bind cell surface and mediate cell migration and adhesion.^7^ Compared with other members of the cathepsin family, relatively little is known about the roles of CTSZ in cancer progression. Analysis of patient samples has revealed a correlation between high CTSZ expression levels and advanced malignancy.^8^, ^9^ In addition, CTSZ has been indicated to affect the epithelial‐to‐mesenchymal transition (EMT) and promote cancer invasion and metastasis.^10^ However, there is no report on the role of CTSZ in CRC development.

In this study, our results indicate that KMT2A is overexpressed in CRC and increasing KMT2A expression is positively correlated with CRC invasion and metastasis. By using both in vitro and in vivo systems, we demonstrate that KMT2A directly influences CRC cell invasion and migration by affecting EMT. We also show CTSZ is one of the important downstream genes of KMT2A. KMT2A can promote CTSZ transcriptional activation through H3K4 trimethylation. Furthermore, besides direct transcription promotion function, p65 is required for the recruitment of KMT2A on CTSZ promoter region. Together, out data provide strong evidences that KMT2A plays a crucial role in CRC metastasis by upregulating CTSZ expression.

## 2 MATERIALS AND METHODS

### 2.1 Cell lines and cultures

HCT116 and DLD1 cell lines were obtained from the Institute of Cell Biology at the Chinese Academy of Sciences (Shanghai, PR China). HCT116 cells were cultured in McCoy's5A medium supplemented with 10% fetal bovine serum (FBS) (Invitrogen), and DLD1 cells were cultured in RPMI1640 medium (Gibco) supplemented with 10% FBS. All cells were cultured in a humidified incubator at 37°C with 5% CO_2_.

### 2.2 RNA extraction, Quantitative real‐time PCR Assays and RNA sequencing

Total RNA from cell lines was isolated using TRIzol reagent (Invitrogen) according to the manufacturer's instructions. Quantitative TaqMan real‐time PCR assays for KMT2A and CTSZ were conducted and all reactions were run in triplicates. After the reactions were complete, the CT values were determined using fixed threshold settings. Data were analyzed by using the 2^−ΔΔCT^ method. Library preparation for both small RNA and mRNA sequencing was performed according to the manufacturer's instructions. Briefly, the total RNA was isolated from HCT116 cells with TRIzol reagent (Invitrogen) and analyzed to determine quantity. High quality total RNA (1 μg) was used as the starting material. Sequencing was performed using HiSeq2500 (Illumina Inc, San Diego, CA) at GeneChem (Shanghai) Co., Ltd.

### 2.3 Knockdown of KMT2A and CTSZ

Cells were seeded onto 6‐well plates at a density of 2 × 10^5^ per well and were given at least 16 hours to allow cell attachment to the well surface before conducting experiments. However, siRNA or negative control was transfected with lipofectamine 2000 (Invitrogen). Two siRNAs were used in the experiments and lentiviral (LV) infection based on siRNAs (LV‐siRNA) was also used to increase the knockdown effect. Here, siRNA and Lentivirus were purchased from GeneChem, Shanghai China and were used according to the manufacturer's instructions (www.genechem.com.cn).

### 2.4 Cell migration and invasion assays

A 24‐well transwell plate was used to examine cancer cell line's migration and invasive abilities. For migration assays, 5 × 10^4^ cells were plated in the top chamber. For invasion assays, chamber inserts were additionally coated with 200 mg/mL of Matrigel and 1 × 10^5^ cells were plated in the top chamber. In both assays, cells were suspended in medium without serum, and medium supplemented with serum was used as a chemo‐attractant in the lower chamber. After incubation at 37°C for 24 hours, the top chambers were wiped with cotton wool to remove the nonmigratory or noninvasive cells. The invading cells on the underside of the membrane were fixed in 4% paraformaldehyde for 15 minutes, airdried, stained, and counted under a microscope.

### 2.5 Western blot assay

The protein concentration was determined through Bradford assay. Cellular proteins were separated by SDS‐PAGE, transferred onto polyvinylidene fluoride membrane and probed with the indicated antibodies. Antibodies that recognize CTSZ (1:500), p65 (1:500), Tubulin (1:500), and GAPDH (1:5000) were obtained from Abcam. Anti‐KMT2A (1:200) was obtained from Sigma‐Aldrich.

### 2.6 Chromatin immunoprecipitation

Cellular proteins were extracted using a modified buffer, followed by immunoprecipitation with the corresponding antibodies. Primers used in qPCR are listed in Supporting Information. For Chromatin immunoprecipitation, the Pierce Agarose ChIP Kit (Thermo) was used. For qPCR followed by chromatin immunoprecipitation (ChIP), the Premix Taq™ Kit (Takara) was used.

### 2.7 Luciferase Assay

CTSZ promoter fragments were amplified and cloned into PGL4 Luciferase Reporter vector (Promega) for the construction of reporter plasmids. Cells were transfected with plasmids using Lipofectamine 2000 for HCT116 cells. 24 hours after transfection, cells were harvested and subjected to the luciferase assay by using Dual‐Luciferase Reporter Assay System (Promega) according to the manufacturer's protocol. Relative light unit is the firefly luciferase activity from the promoter constructs normalized to the corresponding Renilla luciferase.

### 2.8 Tissue specimens

Human cancer samples were collected from 130 patients with CRCs and were included in the analysis. The diagnosis of CRC was confirmed by pathological examination. All clinicopahtological data were collected (Table S1). Immunohistochemical (IHC) staining was employed to analyze the expression levels of KMT2A and CTSZ. The staining intensity of KMT2A and CTSZ was graded on a scale from 0 to 3 (0 for no staining, 1 for weak immunoreactivity, 2 for moderate immunoreactivity, and 3 for strong immunoreactivity) The percentage of immunoreactivity was scored on a scale from 0 to 3 (0 for no positive cells, 1 for <25% of cells positive, 2 for 25%‐50% of cells positive, 3 for 50%‐75%, and 4 for >75 of cells positive). The staining intensity score and the percentage of immunoreactivity score were then multiplied to obtain a composite score (CS; 0, 1, 2, 3, 4, 6, 8, 9 or 12) as described previously.^11^ This study was conducted under the principles of the World Medical Association Helsinki agreement. Ethical approval was obtained from the Ethics Committee of Wenzhou Medical University.

### 2.9 Mouse Model

All mouse experiments were performed according to the regulations of Wenzhou Medical University, and approved by the animal care and use committee of Wenzhou Medical University. Twelve 6‐week‐old male nu/nu mice were divided into two groups (6 mice/group). A small left abdominal flank incision was made, the spleen was exteriorized, and the prepared cells (2 × 10^6^ cells/50 μL/mouse) were injected into the spleen with a 30‐gauge needle. To prevent cell leakage and bleeding, a cotton swab was held over the site of injection for 15 seconds. The injected spleen was returned to the abdomen and the wound was sutured with 6‐0 black silk. Six weeks later, all mice were sacrificed and necropsied for observation of visible metastatic lesions in the liver.

### 2.10 Statistical analysis

All data were expressed as mean ± SD and differences between groups were estimated by Student's *t*‐test or Fisher's exact test at a *P*‐value threshold of 0.05. All statistical analyses were performed with SPSS software version 19.0 (SPSS, Chicago, IL).

## 3 RESULTS

### 3.1 KMT2A expression is associated with CRC progression

**Figure 1:**
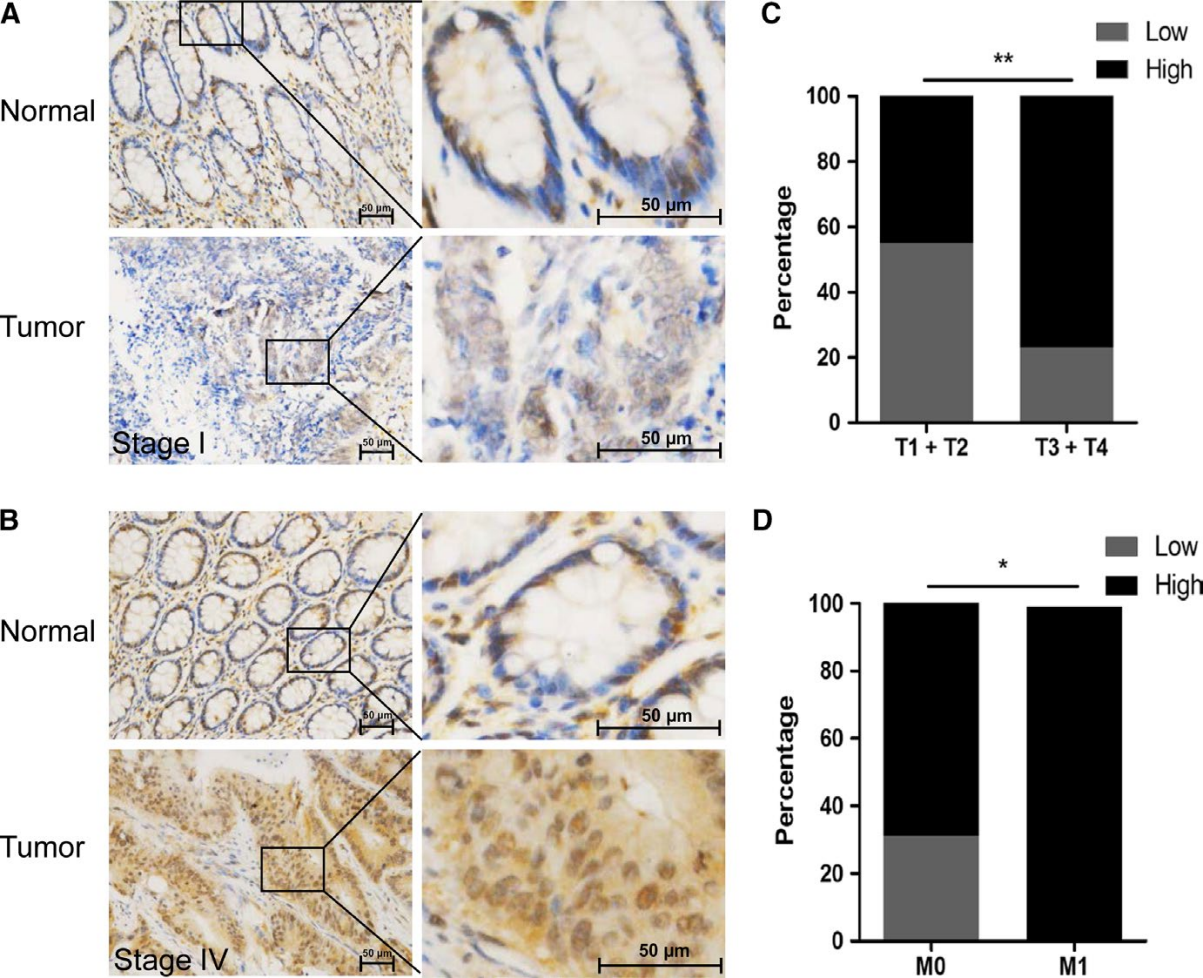
KMT2A expression is associated with colorectal cancer (CRC) development. A and B, The KMT2A expression in human CRC samples. C, The KMT2A expression was correlated with cancer T stage. D, The KMT2A expression was correlated with cancer M stage *P < 0.05, **P < 0.01. Bar, 50 μm

To investigate the role of KMT2A in CRC, we first examined its expression levels in cancer and normal tissues. IHC staining confirmed that KMT2A was overexpressed in CRC in comparison with adjacent normal tissues (Figure 1A). Furthermore, KMT2A expression was positively correlated with tumor invasion (Figure 1B) and metastasis (Figure 1C).

### 3.2 KMT2A promotes CRC cell invasion and metastasis

**Figure 2:**
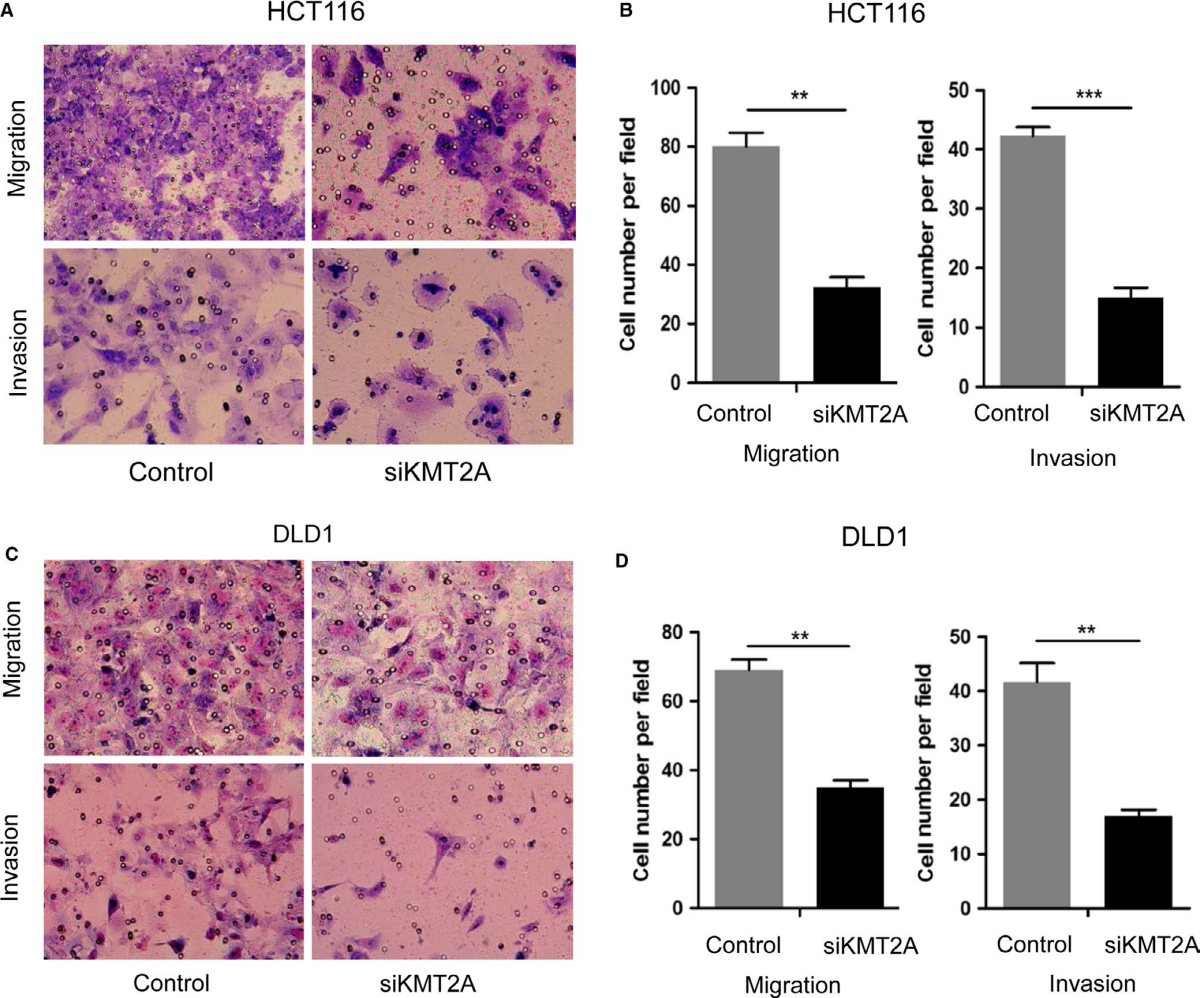
Effect of KMT2A on cancer cell migration and invasion of HCT116 and DLD1 cells. A and B, Inhibition of KMT2A had significant effect on the migration and invasion of HCT116 cells. C and D, Inhibition of KMT2A had significant effect on the migration and invasion of DLD1 cells. Representative fields of invasion (down) or migration (up) cells on the membrane is on the left (magnification of 200×). Average invasion or migration cell number per field is on the right. The invasion or migration cell number of HCT116 and DLD1 was drastically decreased. **P < 0.01, ***P < 0.001

Next, we examined the function of KMT2A on the HCT116 and DLD1 cell invasion and migration (Figure 2). Knockdown of KMT2A caused a significant decrease of migration and invasion abilities in HCT116 cells (Figure 1A,B). Furthermore, the KMT2A silenced DLD1 cells also displayed decreased migration and invasion abilities compared with the control cells (Figure 1C,D).

### 3.3 CTSZ is the key downstream of KMT2A

**Figure 3:**
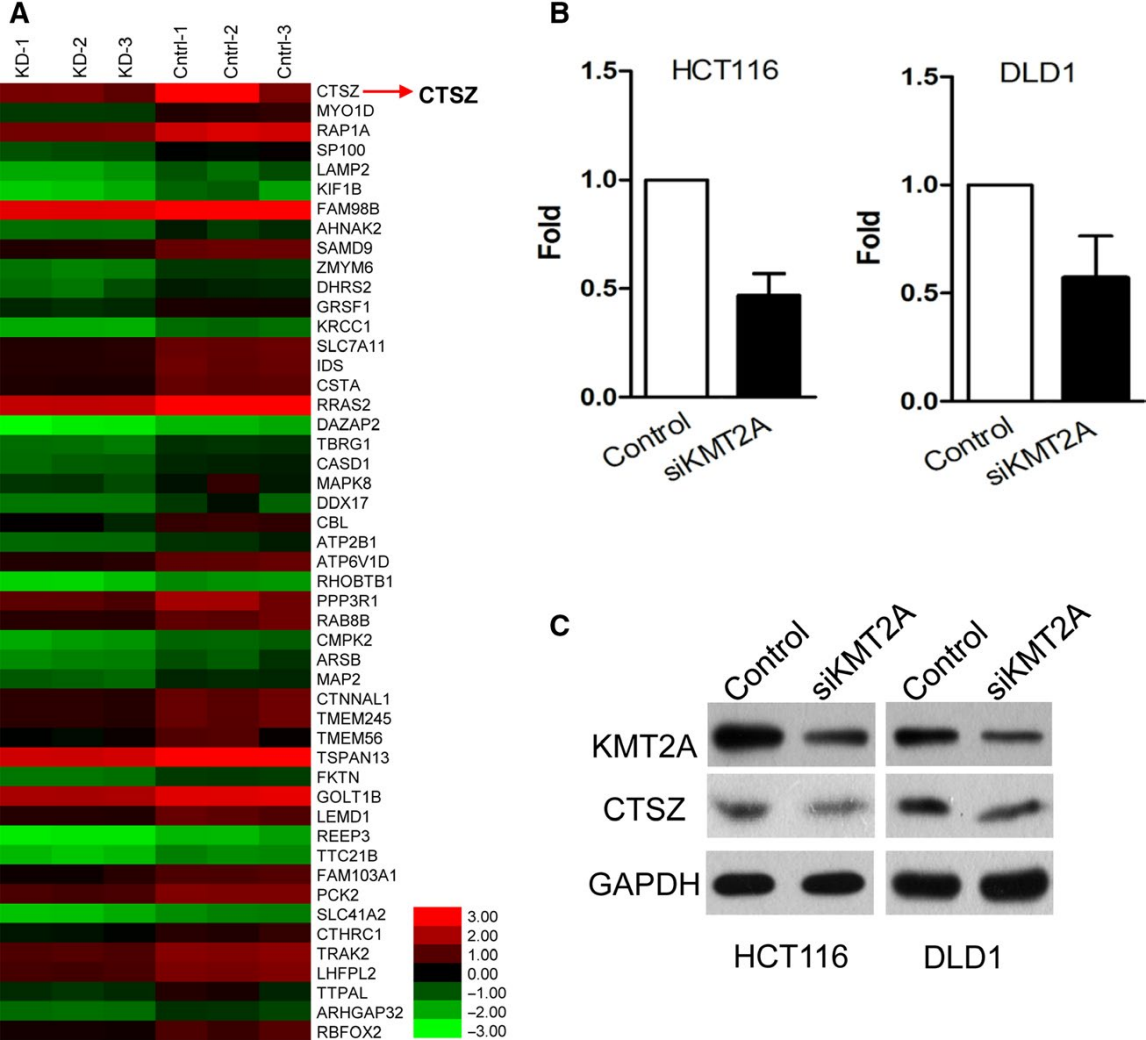
Cathepsin Z (CTSZ) is a downstream gene of KMT2A. A, Microarray analysis showed the differential expression of mRNAs between HCT116 cells with and without KMT2A knockdown. B, CTSZ expression in HCT116 and DLD1 cells after transfection with anti‐KMT2A lenti‐virus detected by PCR. C, CTSZ expression in HCT116 and DLD1 cells after transfection with anti‐KMT2A lentivirus detected by Western blot

To identify the downstream genes of KMT2A, a transcriptome analysis was performed. The differential genes between KMT2A knockdown and control groups were collected (Figure 3A). CTSZ was found to be the most significant down‐regulated gene, followed by KMT2A knockdown. Next, qRT‐PCR and Western blotting assays were employed to verify the transcriptome analysis (Figure 3B,C). The results confirmed that CTSZ is one of the important downstream genes of KMT2A.

### 3.4 CTSZ is associated with CRC progression

**Figure 4:**
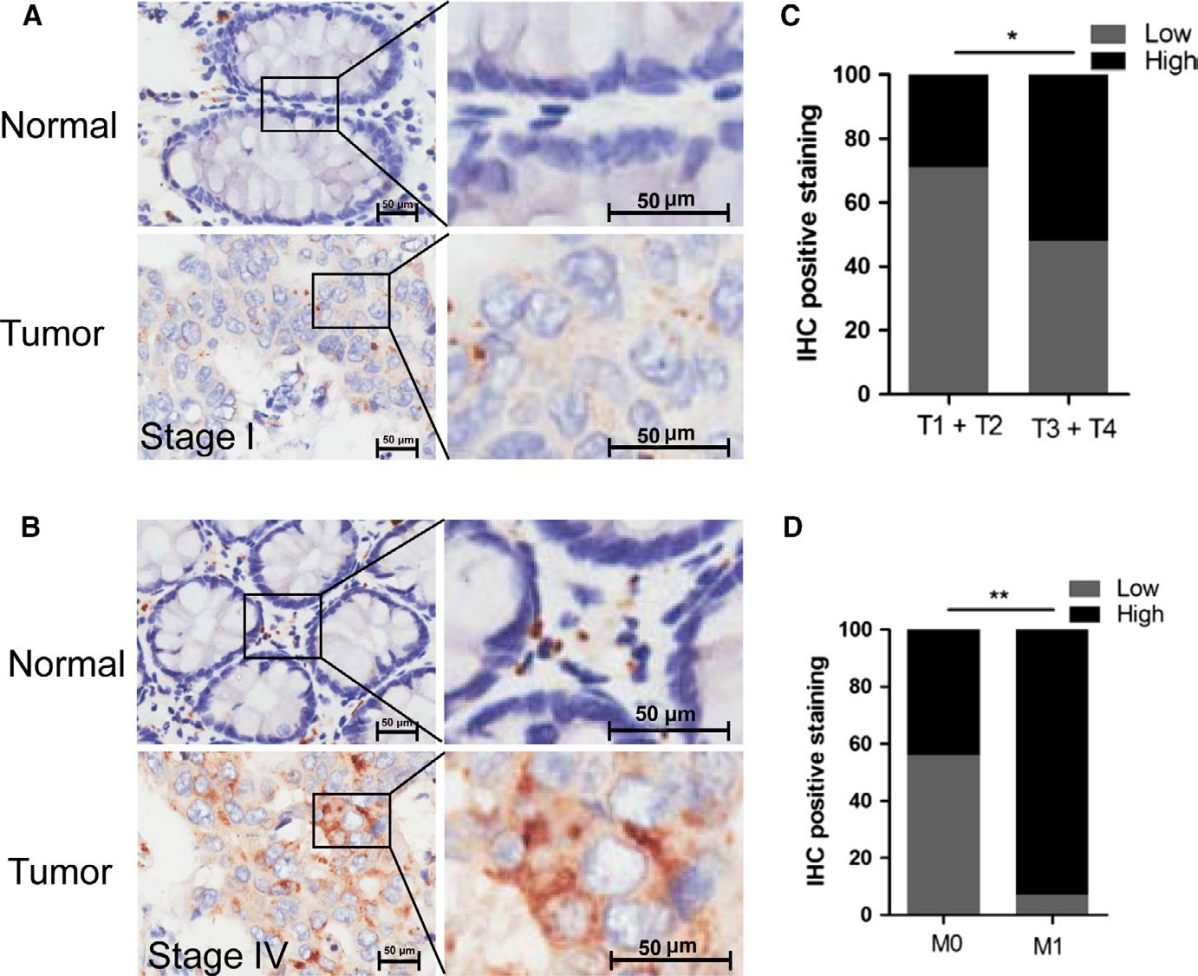
Cathepsin Z (CTSZ) is associated with CRC progression. A and B, The CTSZ expression in human colorectal cancer samples. C, The CTSZ expression was correlated with cancer T stage. D, The KMT2A expression was correlated with cancer M stage *P < 0.05, **P < 0.01. Bar, 50 μm

To confirm the role of CTSZ in CRC, we further determined its expression levels in cancer and adjacent normal tissues. IHC staining confirmed that CTSZ was overexpressed in CRC in comparison with adjacent normal tissues (Figure 4A). Furthermore, KMT2A expression level was positively associated with cancer invasion and metastasis (Figure 4B,C).

### 3.5 Deceased CTSZ inhibits GC cell migration and invasion

**Figure 5:**
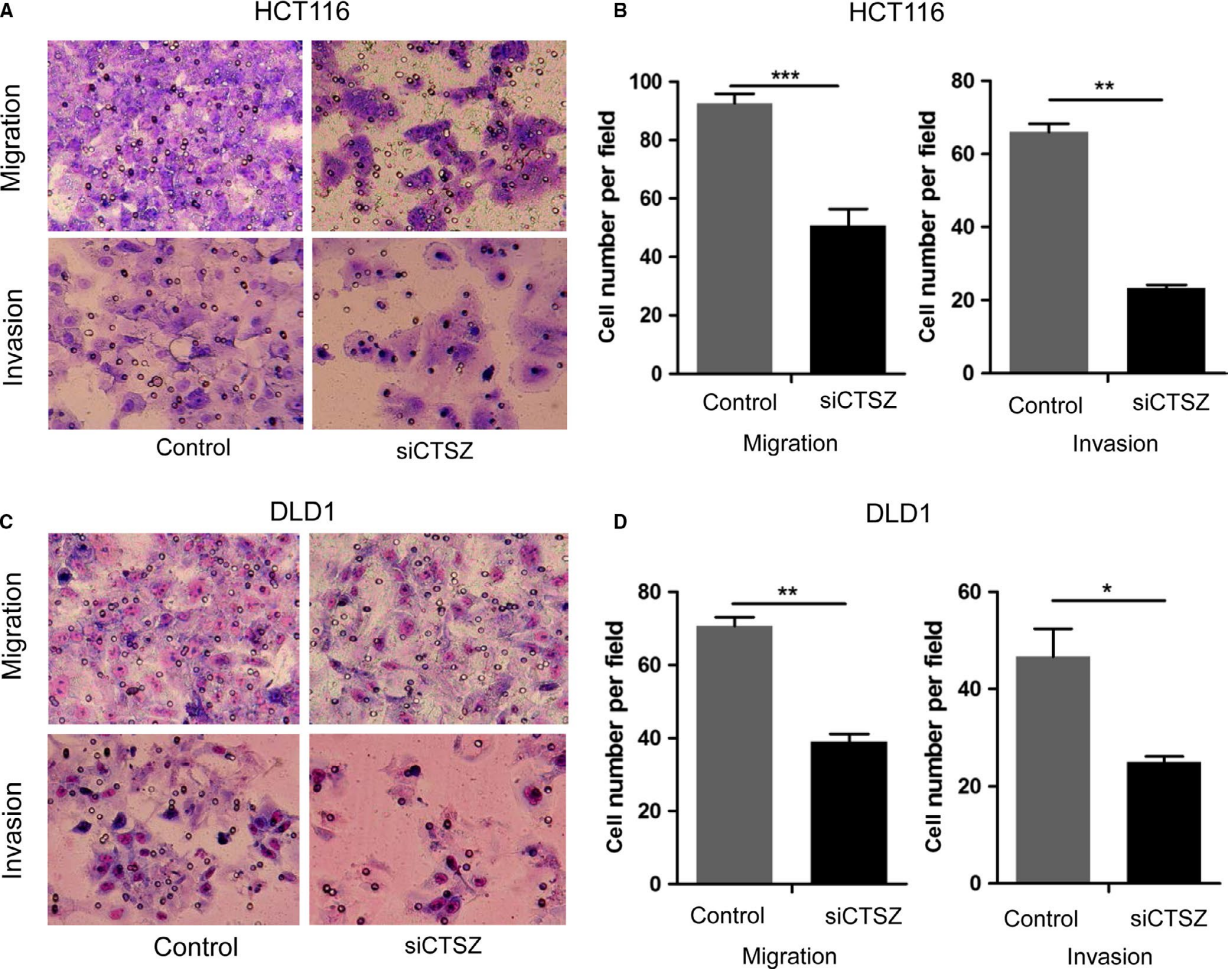
Effect of cathepsin Z (CTSZ) on cancer cell migration and invasion of HCT116 and DLD1 cells. A and B, Inhibition of CTSZ had a significant effect on the migration and invasion of HCT116 cells. C and D, Inhibition of CTSZ had significant effect on the migration and invasion of DLD1 cells. Representative fields of invasion (down) or migration (up) cells on the membrane is on the left (magnification of 200×). Average invasion or migration cell number per field is on the right. The invasion or migration cell number of HCT116 and DLD1 was drastically decreased. *P < 0.05, **P < 0.01, ***P < 0.001

Moreover, we determined the effect of CTSZ on the HCT116 and DLD1 cell invasion and migration. Knockdown of CTSZ caused a significant decrease of migration and invasion abilities in HCT116 cells (Figure 5A,B). In addition, the CTSZ silenced DLD1 cells also displayed decreased migration and invasion abilities compared with the control cells (Figure 5C,D).

### 3.6 KMT2A is recruited by p65 and co‐promotes CTSZ transcriptional activation

**Figure 6:**
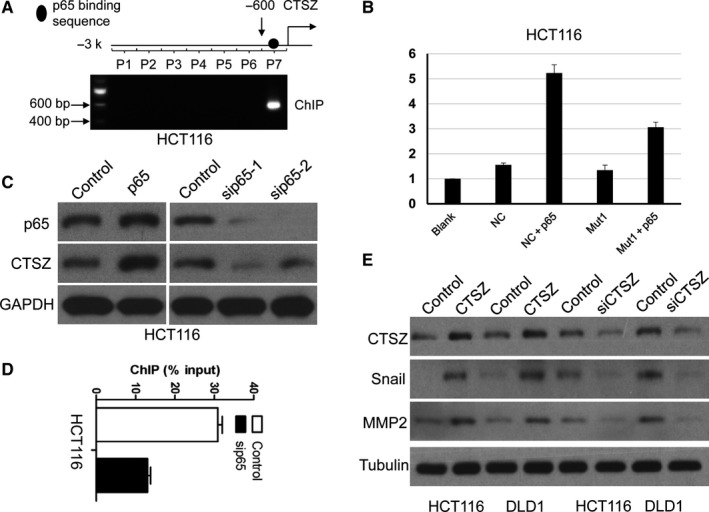
KMT2A is recruited by p65 and co‐promotes cathepsin Z (CTSZ) transcriptional activation. A, A ChIP assay was performed that p65 binding CTSZ promoter region. B, Luciferase assay results showed that KMT2A transactivation of the CTSZ transcription. C, CTSZ expression in HCT116 cells when p65 overexpression and knockdown. D, Knockdown of p65 affected KMT2A binding on CTSZ promoter. E, Alternation of CTSZ levels regulated Snail and MMP2 expression in both HCT116 and DLD1 cell lines

The transcriptional activation of KMT2A depends on transcription factor recruitment on the promoters of downstream genes.^12^ NF‐κB subunit, p65 (RelA), was predicted to bind the promoter of CTSZ and promote CTSZ transcription. A ChIP assay was performed and validated that p65 binds to CTSZ promoter region (Figure 6A). Furthermore, luciferase assay showed that KMT2A transactivation of the CTSZ transcription (Figure 6B). In addition, alternation of p65 levels could regulate CTSZ expression (Figure 6C). Taken together, the above results demonstrated that the transcription factor, p65, can promote CTSZ transcriptional activation and gene expression.

Next, we examined whether p65 could recruit KMT2A on the promoter region of CTSZ. The result of ChIP assay showed that knockdown of p65 could reduce the KMT2A on the promoter of CTSZ (Figure 6D). CTSZ expression can regulate EMT and the expression of matrix metalloproteinases (MMPs).^8^, ^10^ We found that alternation of CTSZ levels could regulate Snail and MMP2 expression in both HCT116 and DLD1 cell lines (Figure 6E).

### 3.7 KMT2A expression is associated with cancer metastasis in vivo

**Figure 7:**
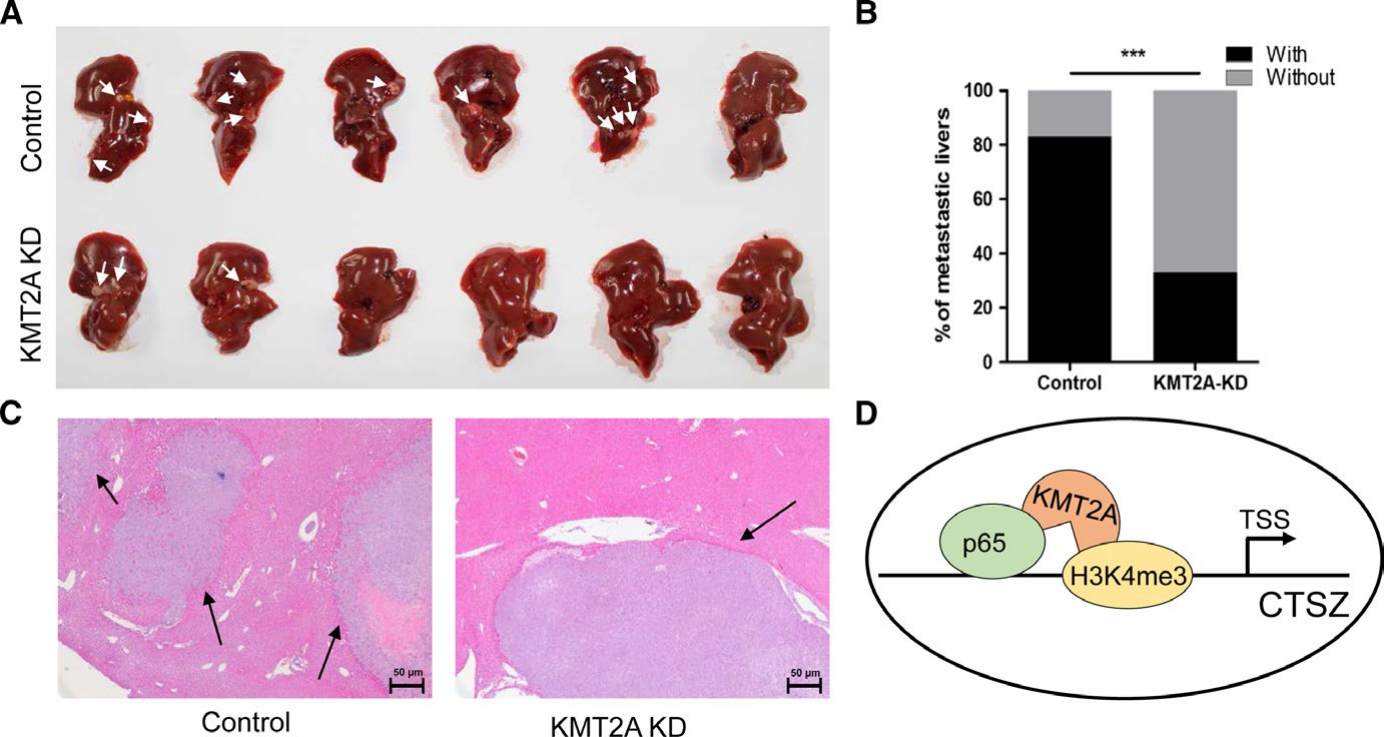
KMT2A controls the colorectal cancer hepatic metastasis in vivo. A and B, The injection of HCT116 cells with inhibited KMT2A into spleen led to decreased information of liver metastasis. C, Hematoxylin and eosin staining (10×) showed that inhibited KMT2A led to decreased information of liver metastasis. D, A schematic diagram of model displaying the implication of KMT2A promoting cathepsin Z (CTSZ) transcriptional activation. ***P < 0.001

To further investigate the function of KMT2A on cancer metastasis in vivo, HCT116 cells with or without KMT2A depletion were injected into the spleens of nude mice. After 6 weeks post‐injection, we found less mice with liver metastasis in KMT2A depletion group (Figure 7A,B). Hematoxylin and eosin assay confirmed that KMT2A depletion led to decreased liver metastasis formation (Figure 7C).

## 4 DISCUSSION

KMT2A has been found to be early drivers of oncogenesis in aggressive lymphoid and myeloid leukemias. The mammalian KMT2A regulates gene transcription through activating H3K4me3 on promoters.^13^ Previous study showed that altered H3K4me3 expression is associated with CRC development.^14^ In the present study, we found that KMT2A was identified to be significantly upregulated in CRC. High level of KMT2A was associated with tumor invasion and metastasis. In addition, KMT2A contributed to CRC cell migration in vitro and vivo. The above results indicated KMT2A as a potential oncogene in CRC. By using microarray screening, we identified and verified that CTSZ was one of the most important downstream genes of KMT2A. Alterations in CTSZ promoter played an important role in enhancing its transcription. In detail, p65 recruited KMT2A and co‐promoted CTSZ expression in CRC cells.

In this study, CTSZ was firstly reported to be upregulated in CRC primary samples. Previously study reported that CTSZ overexpression was involved in cancer progression.^10^ In this study, we further demonstrated that high level of CTSZ was associated with tumor invasion and metastasis. Moreover, CTSZ contributed to invasion and migration of CRC cells. Taken together, these data strongly suggest that CTSZ functions as a proto‐oncogene, and the role of CTSZ as a potential oncogene in CRC development. Furthermore, our data confirmed that CTSZ expression was negatively correlated with E‐cadherin expression and served as driving force to EMT to facilitate CRC progression. Global H3K4me3 is dramatically increased during EMT, the process characterized by the loss of cell adhesion and increased cell mobility.^15^ Thus, we hypothesized that overexpressed KMT2A may increase H3K4me3 and promote CTSZ transcription to increase CTSZ‐mediated EMT and cancer metastasis.

In this study, we identify a new KMT2A/CTSZ nexus that is not reported in CRC. In addition, the KMT2A/CTSZ nexus importantly functions as an oncogenic role in CRC development. Furthermore, p65 is investigated to be critical in this process and mediates KMT2A recruitment and CTSZ transcription (Figure 6). Nevertheless, our data provide important new insights that KMT2A contributes to CRC invasion and metastasis through p65 mediated CTSZ transcription activation.

## CONFLICTS OF INTEREST

The authors declare no conflict of interest.

## DATA AVAILABILITY STATEMENT

The data that support the findings of this study are openly available.

## References

[1] Qamra A , Xing M , Padmanabhan N , et al. Epigenomic promoter alterations amplify gene isoform and immunogenic diversity in gastric adenocarcinoma. Cancer Discov. 2017;7:630‐651. 28320776 10.1158/2159-8290.CD-16-1022

[2] Yang W , Ernst P . SET/MLL family proteins in hematopoiesis and leukemia. Int J Hematol. 2017;105:7‐16. 27796741 10.1007/s12185-016-2118-8

[3] Rao RC , Dou Y . Hijacked in cancer: the KMT2 (MLL) family of methyltransferases. Nat Rev Cancer. 2015;15:334‐346. 25998713 10.1038/nrc3929 PMC4493861

[4] Ballabio E , Milne TA . Molecular and epigenetic mechanisms of MLL in human leukemogenesis. Cancers. 2012;4:904‐944. 24213472 10.3390/cancers4030904 PMC3712720

[5] Hidaka S , Yasutake T , Takeshita H , et al. Differences in 20q13.2 copy number between colorectal cancers with and without liver metastasis. Clin Cancer Res. 2000;6:2712‐2717. 10914715

[6] Turk V , Stoka V , Vasiljeva O , et al. Cysteine cathepsins: from structure, function and regulation to new frontiers. Biochim Biophys Acta. 2012;1824:68‐88. 22024571 10.1016/j.bbapap.2011.10.002 PMC7105208

[7] Akkari L , Gocheva V , Kester JC , et al. Distinct functions of macrophage‐derived and cancer cell‐derived cathepsin Z combine to promote tumor malignancy via interactions with the extracellular matrix. Genes Dev. 2014;28:2134‐2150. 25274726 10.1101/gad.249599.114 PMC4180975

[8] Wang J , Chen L , Li Y , Guan X‐Y . Overexpression of cathepsin Z contributes to tumor metastasis by inducing epithelial‐mesenchymal transition in hepatocellular carcinoma. PLoS ONE. 2011;6:e24967. 21966391 10.1371/journal.pone.0024967 PMC3178578

[9] Vizin T , Christensen IB , Nielsen H , Kos J . Cathepsin X in serum from patients with colorectal cancer: relation to prognosis. Radiol Oncol. 2012;46:207‐212. 23077459 10.2478/v10019-012-0040-0 PMC3472949

[10] Tuo H , Shu F , She S , et al. Sorcin induces gastric cancer cell migration and invasion contributing to STAT3 activation. Oncotarget. 2017;8:104258‐104271. 29262638 10.18632/oncotarget.22208 PMC5732804

[11] Chen T , Li J , Xu M , et al. PKCε phosphorylates MIIP and promotes colorectal cancer metastasis through inhibition of RelA deacetylation. Nat Commun. 2017;8:939. 29038521 10.1038/s41467-017-01024-2 PMC5643311

[12] Liu Y , Zheng X , Yu Q , et al. Epigenetic activation of the drug transporter OCT2 sensitizes renal cell carcinoma to oxaliplatin. Sci Transl Med. 2016;8:348ra97. 10.1126/scitranslmed.aaf3124 27440728

[13] Ford DJ , Dingwall AK . The cancer COMPASS: navigating the functions of MLL complexes in cancer. Cancer Genet. 2015;208:178‐191. 25794446 10.1016/j.cancergen.2015.01.005

[14] Akhtar‐Zaidi B , Cowper‐Sal{middle dot}lari R , Corradin O , et al. Epigenomic enhancer profiling defines a signature of colon cancer. Science. 2012;336:736‐739. 22499810 10.1126/science.1217277 PMC3711120

[15] McDonald OG , Wu H , Timp W , Doi A , Feinberg AP . Genome‐scale epigenetic reprogramming during epithelial‐to‐mesenchymal transition. Nat Struct Mol Biol. 2011;18:867‐874. 21725293 10.1038/nsmb.2084 PMC3150339

