# Supplementary material for: KMT2A histone methyltransferase contributes to colorectal cancer development by promoting cathepsin Z transcriptional activation
Source: Cancer Med. 2019 May 15;8(7):3544–52. doi: 10.1002/cam4.2226 (PMC6601586; doi:10.1002/cam4.2226)
Supplement: Supplementary file 1 [file CAM4-8-3544-s001.docx]

**SUPPLEMENTARY INFORMATION**

**Supplementary Table 1 Clinicopathologic information of 130 CRCs**

| **Variable** | **n, %** |
| --- | --- |
| **Age, y** |  |
| ≤65 | 31, 23.8 |
| >65 | 99, 76.2 |
| **Gender** |  |
| Male | 83, 63.8 |
| Female | 47, 36.2 |
| **Tumor location** |  |
| Colon | 84, 64.6 |
| Rectum | 46, 35.4 |
| **Histology** |  |
| Adenocarcinoma | 121, 93.1 |
| Mucinous | 9, 6.9 |
| **Tumor invasive depth** |  |
| T1-T2 | 20, 15.4 |
| T3-T4 | 110, 84.6 |
| **Lymph node status** |  |
| N0(n=0) | 77, 59.2 |
| N1(n≤3) | 35, 26.9 |
| N2(n>3) | 18, 13.8 |
| **Tumor Metastasis** |  |
| M0 | 111, 85.4 |
| M1 | 19, 14.6 |

**Supplementary Table 2 CTSZ primers for ChIP assay.**

| Primer 1 | FORWARD | TGACCATCTCACCCCTCCTCC |
| --- | --- | --- |
|  | REVERSE | TTCCTGTAGCATGGGGAGAGCTG |
| Primer 2 | FORWARD | GCCTGCCTTGCTGACCATGC |
|  | REVERSE | CTTGCCAGGTCATCAGGTGGC |
| Primer 3 | FORWARD | GCTGTAGAATGTGCTGTTGGACTTCAC |
|  | REVERSE | CCAAGCTTGAGCTGGCGGTG |
| Primer 4 | FORWARD | TTATGCCCAGAGGTTGGGAGGC |
|  | REVERSE | CAGTGGCTGCCTCCTGACTT |
| Primer 5 | FORWARD | CAGCCTTGCTCTCCACTCGG |
|  | REVERSE | GGCTTCTCGCTCCCTGCG |
| Primer 6 | FORWARD | ATGCCGGGCCCTGTTCTTAC |
|  | REVERSE | AAGGGATCCTGGGATGCGG |
| Primer 7 | FORWARD | AAGGATCCCTCCTTCCTTCTCTGC |
|  | REVERSE | GGCCCCGCGCCGGCTCCT |
| Mutagenesis | CTSZ mut-1 | GGCCCAGATGTGAGGCTGGGGGATTCTATCCAGGGCTCAGGGCCCCAGG |
|  | CTSZ mut-2 | CCTGGGGCCCTGAGCCCTGGATAGAATCCCCCAGCCTCACATCTGGGCC |
|  | CTSZ mut-3 | GGGACAGCCCCGCCCCGGGATAGTGTATCGGCCTGGCCGCCGCAGCCTTAAG |
|  | CTSZ mut-4 | CTTAAGGCTGCGGCGGCCAGGCCGATACACTATCCCGGGGCGGGGCTGTCCC |
